# p31^comet^ inactivates the chemically induced Mad2-dependent spindle assembly checkpoint and leads to resistance to anti-mitotic drugs

**DOI:** 10.1186/2193-1801-2-562

**Published:** 2013-10-25

**Authors:** Toshiyuki Habu, Tomohiro Matsumoto

**Affiliations:** Radiation Biology Center, Kyoto University, Yoshida-Konoe cho, Sakyo ku, Kyoto, Japan

**Keywords:** p31^comet^, Mad2, Anti-mitotic drug, Spindle assembly checkpoint, Drug resistance

## Abstract

Mad2 is a key component of the spindle assembly checkpoint (SAC) that delays the onset of anaphase until all kinetochores are attached to the spindle. It binds to Cdc20 and prevents it from promoting destruction of an anaphase inhibitor, Securin. Previously, we showed that a Mad2-binding protein, p31^comet^, formed a complex with Mad2 upon the completion of spindle attachment. Here, we showed that the overexpression of p31^comet^ can abolish the Mad2-dependent SAC that is induced by anti-mitotic drugs, including nocodazole, taxol, and monastrol; these drugs, except monastrol, cause aneuploidy in HeLa cells. In the absence of Eg5, which is a target of monastrol, overexpression of p31^comet^ caused premature destruction of Securin and premature sister chromatid separation, but it did not cause aneuploidy. These results indicated that Eg5 kinesin function might be required for checkpoint exit and mitotic progression. Moreover, overexpression of p31^comet^ led to resistance against apoptosis that was induced by nocodazole and taxol in human cells, and taxol resistance was dependent on the p31^comet^/Mad2 protein expression level ratio of in cancer cell lines. These results indicated that p31^comet^ is an indicator of resistance to anti-mitotic drugs in cancer cells.

**Electronic supplementary material:**

The online version of this article (doi:10.1186/2193-1801-2-562) contains supplementary material, which is available to authorized users.

## Background

The spindle assembly checkpoint (SAC) is a surveillance mechanism that delays the onset of anaphase until the completion of the spindle microtubule attachment to all kinetochores during mitosis (Elledge 1998; Kops et al. 2005; Nasmyth 2005). Mad2 is a master regulator of the checkpoint and is found in a complex with its target, Cdc20, which is a co-activator of anaphase promoting complex or cyclosome (APC/C), from prometaphase to metaphase. The association and inhibition of Cdc20 by SAC to delay the proteolysis of Securin and cyclin B_1_, appears to be a central process in the signaling SAC cascade (Fang et al. 1998; Hwang et al. 1998; Kim et al. 1998; Shah and Cleveland 2000). Mad2 has two conformations, 'open’ (O-Mad2) and 'close’ (C-Mad2) (Luo et al. 2004; Mapelli et al. 2007). The Mad1-C-Mad2 complex acts as a template and recruits O-Mad2 to convert Mad2 molecules into Cdc20 inhibitors (C-Mad2-Cdc20) (Musacchio and Salmon 2007). C-Mad2-Cdc20 binds to the BubR1-Bub3 complex, forming the mitotic checkpoint complex (MCC). By binding to APC/C, the MCC inhibits the ubiquitylation activity onto Securin and Cyclin B_1_ (Sudakin et al. 2001).

Upon the completion of spindle attachment, Mad2 diminishes from the kinetochores. The microtubule-kinetochore interaction is thought to silence SAC signal. p31^comet^, which we identified by the yeast two-hybrid system as a human Mad2-binding protein, is one of the candidates for the silencer of SAC (Habu et al. 2002). We showed that the formation of the p31^comet^-Mad2 complex coincides with the dissociation of Mad2 from Cdc20, and the overexpression of p31^comet^ abolishes the SAC function in a Mad2-Cdc20 complex-dependent manner. Additionally, p31^comet^ can bind to only the Cdc20-bound conformation of Mad2 (C-Mad2), and can block the biochemical function of C-Mad2 *in vitro* (Xia et al. 2004) and can compete with O-Mad2. From these observations, it has been proposed that p31^comet^ acts as an inhibitory cap on the Mad2-C-Mad2 complex (Mapelli et al. 2006; Musacchio and Salmon 2007). In addition to the model, it has been proposed that p31^comet^ contributes to SAC silencing by promoting Cdc20 ubiquitylation, leading to the disassembly of the MCC (Jia et al. 2011; Varetti et al. 2011). p31^comet^ also promotes the dissociation of Cdc20 from BubR1 in an ATP-dependent manner (Teichner et al. 2011), and this dissociation is co-related to Cdc20 phosphorylation (Miniowitz-Shemtov et al. 2012). Recent studies showed that p31^comet^ promotes an early step in MCC disassembly, extracting Mad2 and leaving behind a BubR1-Bub3-Cdc20 complex (Hagan et al. 2011; Westhorpe et al. 2011). Taken together, p31^comet^ plays a role in silencing the Mad2-dependent SAC.

The chemical inhibitors of mitotic spindle microtubules are commonly used for cancer therapy, and experimental approaches to observe spindle function, and mitosis studies (Mayer et al. 1999; Kapoor et al. 2000; Skoufias et al. 2001; Sudo et al. 2004; Tao et al. 2005; Shi et al. 2008). Nocodazole and vinca alkaloids accelerate microtubule depolymerization and therefore generate unattached kinetochores. Taxol interferes with microtubule dynamics, though the sister kinetochores are closer together and remain bound to microtubules. These observations indicate that these agents generate a loss of kinetochore tension. In contrast, monastrol and KSP-IA are inhibitors of Eg5, which is a mitotic spindle motor protein belonging to the kinesin superfamily. Eg5 is required for centrosome separation and the formation of bipolar spindle in mitosis; therefore, inhibition of Eg5 causes mitotic arrest with the monopolar spindles (Kapoor et al. 2000). Monastrol also reduces inter-kinetochore tension because many attachments in the monopolar spindles are syntelic.

To examine p31^comet^ function in human cells, drug treatment approaches to activate SAC were used. The overexpression of p31^comet^ could abolish the nocodazole and taxol-induced SAC and results in aneuploid cells, but the monastrol-induced SAC does not result in aneuploidy although Securin destruction was observed. HeLa cells whose Eg5 kinesin has been depleted by RNA interference (RNAi) caused the Mad2-dependent mitotic arrest similar to monastrol treatment. When p31^comet^ was overexpressed in Eg5-depleted cells, the cells arrested in mitosis with the same kinetics as Eg5-depleted cells, despite the destruction of Securin and the dissociation of sister chromatids. These results indicated that the overexpression of p31^comet^ could overcome drug-induced Mad2-dependent SAC activation, and it might catalyze Mad2 inactivation during mitosis. Furthermore, the overexpression of p31^comet^ induced resistance to apoptosis that was induced by nocodazole and taxol in human cancer cells independent on p53 function. The expression level of p31^comet^ protein in various cancer cell lines was observed, and the ratio of p31^comet^/Mad2 protein expression levels correlated with taxol sensitivity. These results may indicate a model to explain the roles of SAC and aneuploidy in tumorigenesis.

## Results

### p31^comet^ binding to Mad2 protein

**Figure 1:**
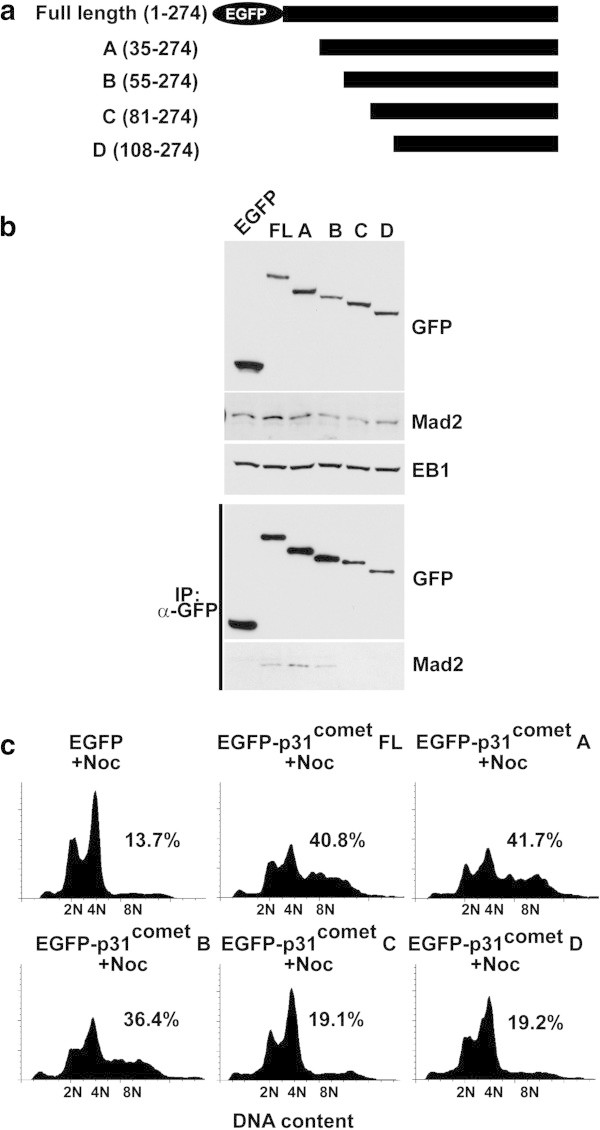
**p31** ^**comet**^ **binding to Mad2 protein. (a)** Diagrams of N-terminal deletion p31^comet^ fragments. **(b)** p31^comet^ binding to Mad2 protein. EGFP, EGFP-tagged full-length p31^comet^, and a series of N-terminal deletion EGFP-tagged p31^comet^ were overexpressed in HeLa cells by adenovirus infection and the expression level of each protein was monitored by western blotting with anti-GFP antibody, using anti-Mad2 and anti-EB1 antibodies for loading controls. The cell extracts overexpressing each protein were prepared for immunoprecipitation with anti-GFP antibody, followed by western blotting with anti-Mad2 and anti-GFP antibodies. **(c)** Fluorescence-activated cell sorting (FACS) analysis of the cells overexpressing EGFP-tagged full-length p31^comet^ and a series of N-terminal deletion EGFP-tagged p31^comet^ in the presence of nocodazole (100 ng/mL). A population of >4 N DNA content in the cells was shown.

To verify the binding to Mad2 protein, series of p31^comet^ fragments tagged with EGFP was overexpressed in HeLa cells (Figure 1a and b), and immunoprecipitation was performed with anti-GFP antibody. As our previous study using a yeast two hybrid assay showed (Habu et al. 2002), Mad2 protein was immunoprecipitated with full-length, A, and B fragments of p31^comet^ but not with C and D fragments (Figure 1b). The position between amino acids 55 and 81 of p31^comet^ might be responsible for binding to Mad2 protein. Full length p31^comet^ can abolish the SAC function in the presence of nocodazole in a Mad2-dependent manner (Habu et al. 2002). The same assay was performed in the presence of nocodazole using a series of p31^comet^ fragments (Figure 1c). When overexpressed, cells with 8 N DNA content were observed in fluorescence-activated cell sorting (FACS) analysis with the same kinetics with fragments (full-length, A, B) that could bind to Mad2 *in vivo* (full-length: 40.8%, A: 41.7%, and B: 36.4%), but the same levels were not observed for fragments C and D, and EGFP only (C: 19.1%, D: 19.2%, and EGFP: 13.7%), which could not bind to Mad2 protein. An amino-terminal fragment of p31^comet^ (1-80 amino acids), which could bind to Mad2 using a yeast two-hybrid assay (Habu et al. 2002), also could override nocodazole-induced SAC (Additional file 1: Figure S1). In addition to these data, L76A/L77A mutant, in which was located in deleted region in fragment C of p31^comet^, could not override SAC (Additional file 1: Figure S1). These data indicated that p31^comet^-direct binding to Mad2 protein might be important for SAC function.

### The effect of antimitotic drugs in p31^comet^ overexpressing cells

**Figure 2:**
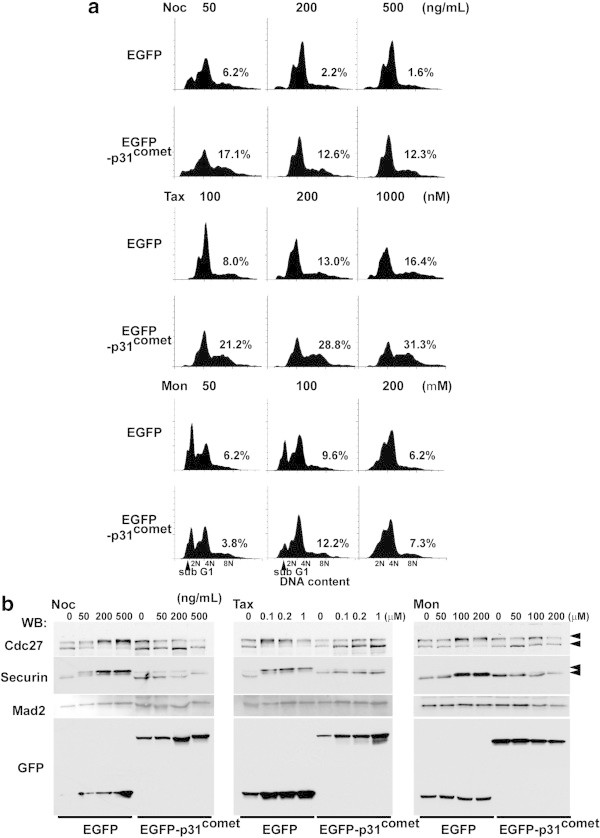
**The effect of p31** ^**comet**^ **overexpression on anti-mitotic drug-treated cells. (a)** FACS analysis of p31^comet^-overexpressing cells treated with anti-mitotic drugs (nocodazole, taxol, and monastrol). Cells were infected with EGFP or EGFP-p31^comet^ adenovirus for 24 h, washed with fresh medium twice, and treated with anti-mitotic drugs at the indicated concentration for 24 h. These cells were prepared for FACS analysis. A population of >4 N DNA content in the cells was shown. **(b)** Western blotting analysis of p31^comet^-overexpressing cells treated with anti-mitotic drugs. Drug-treated cell extracts were subject to western blotting with anti-Cdc27 and anti-Securin antibodies to monitor the cell cycle status, anti-GFP antibody for EGFP and EGFP-p31^comet^, and anti-Mad2 antibody for a loading control.

Antimitotic drugs (nocodazole, taxol, and monastrol) were used to observe SAC function *in vivo* because each drug shows a different effect on spindle morphology and checkpoint machinery. To test the effect of each drug in p31^comet^ overexpressing cells, the cells infected with EGFP or EGFP- p31^comet^ adenoviruses were treated with serial dilutions of each drug as indicated in Figure 2a, and incubated for 24 h. The cell cycle progression of these cells was analyzed by FACS, and the mitotic status was monitored by detecting the accumulation of Securin and the phosphorylation of Cdc27 protein (Tugendreich et al. 1995; Zou et al. 1999). When the cells overexpressing p31^comet^ were treated with nocodazole, the overexpression of p31^comet^ led the appearance of cell fraction with 8 N DNA content (12.3-17.1%), but this was not observed with the overexpression of EGFP (1.6-6.2%) (Figure 2a). Depending on the dosage of nocodazole, the cell fraction with 8 N DNA content decreased. In the treatment with taxol, the overexpression of p31^comet^overcame the taxol-induced SAC (EGFP-p31^comet^: 21.2-31.3%, EGFP: 8-16.4%) (Figure 2a). With overexpressed p31^comet^, no accumulation of Securin, and the decreased phosphorylation levels of Cdc27 protein were observed with the nocodazole treatment (Figure 2b). In contrast, the monastrol-treatment induced accumulation in mitosis, which is Mad2-dependent mitotic arrest (Additional file 1: Figure S2), and differences in the fraction of 8 N DNA contents and cell cycle profiles between EGFP and EGFP-p31^comet^ overexpression were not observed by FACS analysis, although the sub-G1 fraction was partially suppressed in EGFP-p31^comet^ overexpressing cells (Figure 2a, lower panels). In HeLa cells, the low-dosage monastrol treatment induced apoptosis. From western blotting analysis, no accumulation of Securin, and the decreasing phosphorylation level of Cdc27 protein were observed in these cells with the nocodazole treatment despite the accumulation in mitosis that was observed by FACS analysis of p31^comet^ overexpressing cells (Figure 2a and b). To confirm the monastrol effect in p31^comet^ overexpressing cells, the cells that were treated with different doses of nocodazole for 6 h were incubated in monastrol containing medium (50 nM) for 18 h (Additional file 1: Figure S3). In this case, the accumulation of G_2_/M fraction cells was also observed in the cells overexpressing EGFP and EGFP-p31^comet^. Nocodazole treatment of monastrol-pretreated cells was also examined. With this treatment, the accumulation of the cells with 4 N DNA contents was observed as it was with a single round of monastrol treatment (data not shown). These data indicated that p31^comet^-overexpression could inactivate SAC that is induced by the antimitotic drugs nocodazole and taxol but not monastrol.

### Localization of Mad2 and microtubule array of in p31^comet^ overexpressing cells treated with antimitotic drugs

**Figure 3:**
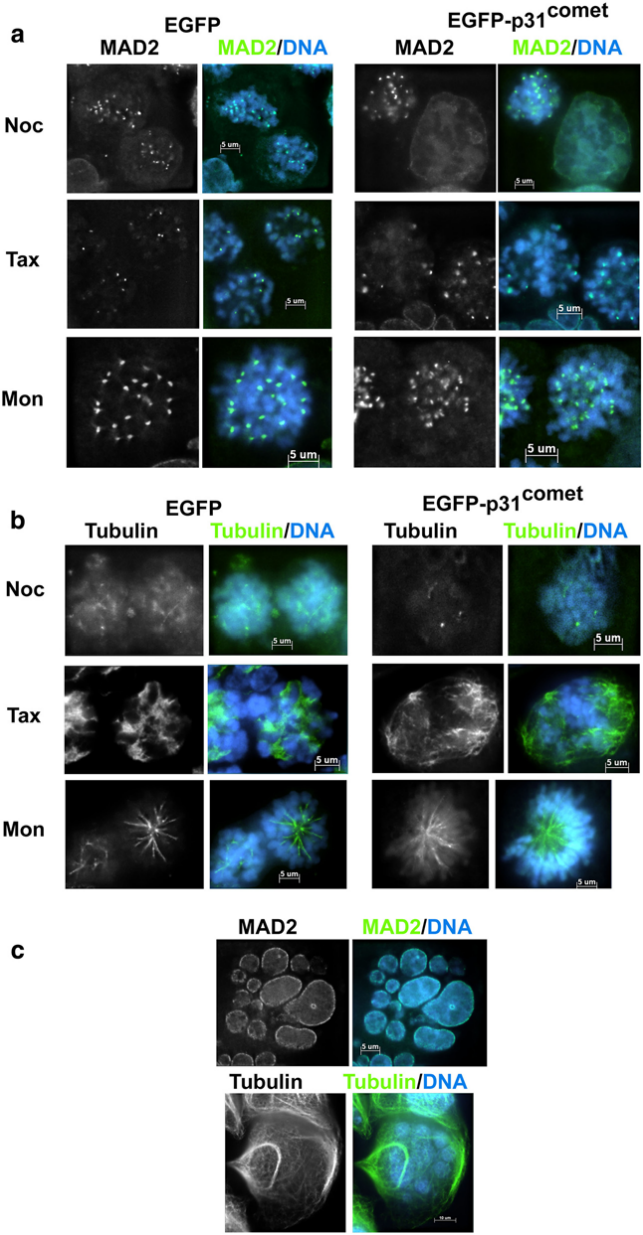
**Localization of Mad2 and microtubule arrays of in p31** ^**comet**^ **-overexpressing cells in the presence of anti-mitotic drugs. (a)** Mad2 localization in p31^comet^ overexpressing cells. HeLa cells stably expressing EGFP-Mad2 were infected with EGFP or EGFP-p31^comet^ adenovirus for 24 h, washed with fresh medium twice, and treated with anti-mitotic drugs at the indicated concentration for 24 h described in Figure 2. Cells were fixed with 3% paraformaldehyde and counterstained with Hoechest33324 for DNA (shown in blue). EGFP-Mad2 was shown in green. Cells were treated with the following drugs: (upper panels) nocodazole, (middle panels) taxol, and (lower panels) monastrol. EGFP (left panels) or EGFP-p31^comet^-expressing (right panels) cells were shown. **(b)** Array of microtubules in p31^comet^-overexpressing cells. HeLa cells were infected with EGFP or EGFP-p31^comet^ adenovirus for 24 h, washed with fresh medium twice, and treated with anti-mitotic drugs at the indicated concentration for 24 h. Cells were fixed with cold methanol, stained with β-tubulin antibody for microtubules (shown in green), and counterstained with Hoechest33324 for DNA (shown in blue). Cells were treated with the following drugs: (upper panels) nocodazole, (middle panels) taxol, and (lower panels) monastrol. EGFP (left panels) or EGFP-p31^comet^-expressing (right panels) cells were shown. *Note*: EGFP and EGFP-p31^comet^ signals were not detectable in this condition. **(c)** Array of microtubules and Mad2 localization in p31^comet^ overexpressing cells. HeLa cells were infected with EGFP-p31^comet^ adenovirus for 24 h and, treated with Taxol for 24 h. Cells were fixed with cold methanol, stained with Mad2 (upper panel) or α-tubulin (lower panel) antibodies (shown in green), and counterstained with Hoechest33324 for DNA (shown in blue).

Because p31^comet^-overexpression inactivates SAC that are induced with antimitotic drugs, we observed the localization of Mad2 and the array of microtubules in the cells. HeLa cells stably expressing EGFP-tagged Mad2 were treated with EGFP or EGFP-p31^comet^ adenoviruses and were exposed to the antimitotic drugs. In p31^comet^-overexpressing cells treated with nocodazole, the signals of EGFP-Mad2 on unattached kinetochores were detectable as EGFP-overexpressing cells (Figure 3a, upper panels) in mitosis, but the signals were diffused into the cytoplasm in enlarged giant cells (Figure 3a, upper right panel), which appeared predominantly in EGFP- p31^comet^ overexpressing cells. In the cells treated with taxol, the signals of EGFP-Mad2 on unattached kinetochores were detectable as EGFP-overexpressing cells, although a lower number of signals was detectable compared with the cells treated with nocodazole, which was also reported in a previous study (Figure 3) (Skoufias et al. 2001). Taxol treatment p31^comet^overexpressing cells induced multinucleated cells rather than aneuploid cells (Figure 3a-c). To address aneuploidy and/or multinuclei in p31^comet^-overexpressing cells, chromosome spread analysis was performed. Although well isolated mitotic condensed chromosomes were observed in EGFP-overexpressing cells, no mitotic chromosomes were observed in p31^comet^-overexpressing cells because there was no mitotic arrest (Additional file 1: Figure S4a and b). In contrast, the cells arrested at prometaphase in both EGFP and EGFP-p31^comet^ overexpressing cells, when the cells were exposed to monastrol (Figure 3a and b). The signals of EGFP-Mad2 were detected on one sister kinetochore in the majority of the control cells as reported (Figure 3a, lower, left panel). When p31^comet^ was overexpressed in the cells, the signals seemed to be positive on both sister kinetochores (Figure 3a, lower, right panel). Based on statistical analysis, 47% of arrested cells were positive on both sister kinetochores for Mad2 (n = 55), compared with 16% in EGFP overexpressing cells (n = 35).

Next, immunostaining with α-tubulin antibody was examined in p31^comet^ overexpressing cells treated with these drugs. The array of microtubules was not detectable in both EGFP and p31^comet^-overexpressing cells with nocodazole treatment because of the depolymerization of the microtubules (Figure 3b). The taxol treatment increased the stability of microtubules; therefore, well-bundled microtubules were observed in interphase and/or mitotic cells overexpressing both EGFP and p31^comet^ (Figure 3b and c). Because the taxol treatment causes centrosome amplification (Wang et al. 2000), it resulted in multinucleated cells in p31^comet^ overexpressing cells rather than giant cells (Figure 3a-c, Additional file 1: Figure S4). In contrast, when the cells were exposed to monastrol, monoasters array of microtubules was observed using the p31^comet^ overexpression as the control (Figure 3b), although Mad2 seemed to localize on both sister kinetochores. This indicated that p31^comet^ overexpression caused SAC inactivation in the presence of nocodazole or taxol, but it did not affect Mad2 localization or microtubule arrays and led to aneuploid or multinucleated cells. As observed in monastrol-treatment experiments, p31^comet^ could inactivate SAC and caused premature destruction of Securin in spite of Mad2 kinetochore localization, although cell cycle arrest during mitosis.

### The SAC inactivation activity of p31^comet^ in Eg5-depleted cells

**Figure 4:**
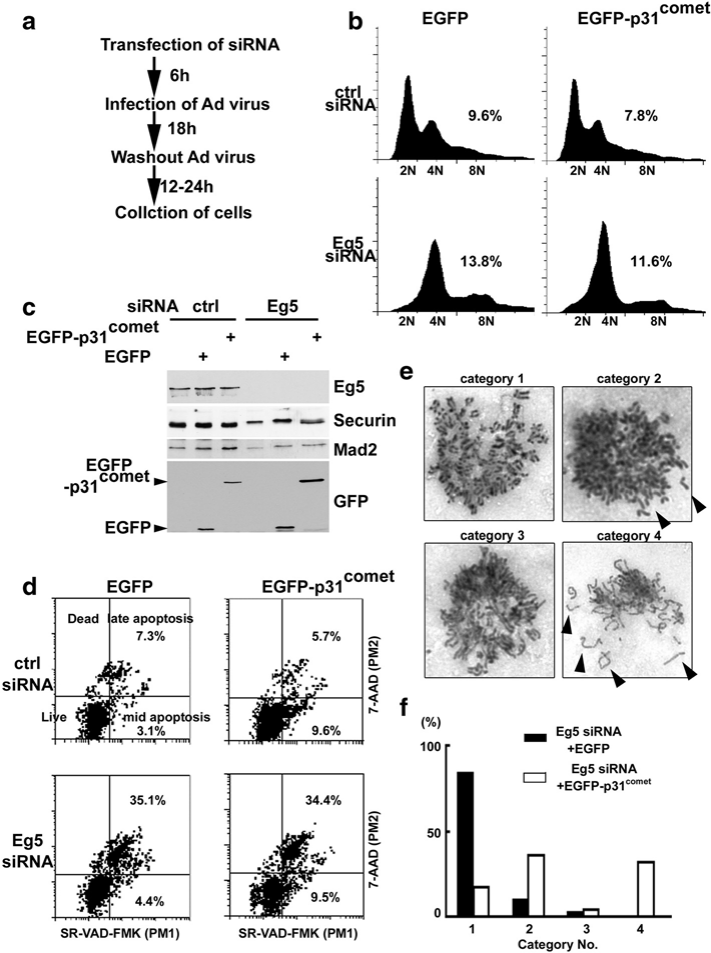
**The effect of p31** ^**comet**^ **overexpression in Eg5-depleted cells. (a)** Scheme of p31^comet^ overexpression in Eg5-depleted cells. **(b)** FACS analysis of p31^comet^-overexpressing cells with Eg5-depletion. Cells treated with Eg5 siRNA or control siRNA were infected with EGFP or EGFP-p31^comet^ adenovirus and prepared for FACS analysis. A population of >4 N DNA content in the cells was shown. **(c)** Western blotting analysis of p31^comet^-overexpressing cells with Eg5-depletion. The siRNA-treated cell extracts were subject to western blotting with anti-Eg5 antibody to monitor Eg5 depletion, anti-Securin antibody to monitor cell cycle status, anti-GFP antibody for EGFP and EGFP-p31^comet^, and anti-Mad2 antibody for a loading control. **(d)** Apoptosis analysis of Eg5-depleted cells. Eg5-depleted cells overexpressing EGFP or EGFP-p31^comet^ were subject to apoptosis analysis with Multicaspase detection kit. A population of apoptotic cells in each treatment was shown. **(e, f)** Chromosome analysis of p31^comet^-overexpressing cells with Eg5-depletion. The arrested cells were fixed in methanol/acetone solution and spread onto glass slides. The chromosome spreads were stained with Giemsa dye and observed with microscopy. Typical chromosome morphologies were shown in **(e)** and categorized into four types. Fifty chromosome spreads were observed and scored.

p31^comet^ overexpression could not overcome mitotic arrest in monastrol treated cells like it could in nocodazole and taxol treated cells. Although the destruction of Securin protein and the phosphorylation of Cdc27 were observed in p31^comet^-overexpressing cells treated with monastrol, the cells showed a prolonged arrest. Because Eg5 kinesin is the target of monastrol, p31^comet^ was overexpressed in cells, in which Eg5 was depleted with siRNA (Figure 4a). As shown in the control experiment in Figrue 4b, Eg5-depletion with siRNA induced G_2_ or mitotic arrest similar to monastrol-treatment 36 h after transfection, and the depleted cells were showed evidence of apoptotic cell death after 42 h (Figure 4d, lower left panel). Western blotting was performed to confirm the Eg5-depletion (Figure 4c, lanes 4-6), and over 90% of Eg5 protein were depleted with this siRNA compared to control siRNA (Figure 4c, lanes 1-3). When p31^comet^ was overexpressed in the Eg5-depleted cells, the cells arrested in mitosis like cells with EGFP overexpression, and cells with 8 N DNA content were not observed with monastrol-treatment (Figure 4b). Forty-two hours after transfection, p31^comet^ overexpression in the Eg5-depleted cells resulted in apoptosis with similar kinetics as EGFP overexpression (Figure 4d, lower panels). Although the Eg5-depleted cells overexpressing p31^comet^ arrested in mitosis, the protein level of Securin was lower than that of the control cells overexpressing EGFP-p31^comet^ with monastrol treatment (Figure 4c, lanes 5 and 6). Next, chromosome spread analysis was used to examine the Eg5-depleted HeLa cells overexpressing EGFP or p31^comet^ (Figure 4e and f). We categorized the chromosome spreads into four different groups (Figure 4e) and measured the numbers 36 h after transfection (Figure 4f). Interestingly, the overexpression of p31^comet^ caused premature release of sister chromatid cohesion (Figure 4e, category 2 and 4), but the overexpression of EGFP did not (Figure 4e, category 1). Forty-one percent of cells (Figure 4e, category 2) and 38% of cells (Figure 4e, category 4) showed premature separation of sister chromatid; strikingly, chromosomes of category 3 and 4 were elongated compared with those of category 1 and 2. The partial decondensation might begin in the p31^comet^overexpressing cells with depleted Eg5 after the inactivation of SAC. These data indicated that p31^comet^ inactivated SAC, but progression to next cell cycle did not occur in the absence of Eg5 function, which was similar to monastrol treatment.

### Resistance to anti-mitotic drugs in p31^comet^ overexpressing cells

**Figure 5:**
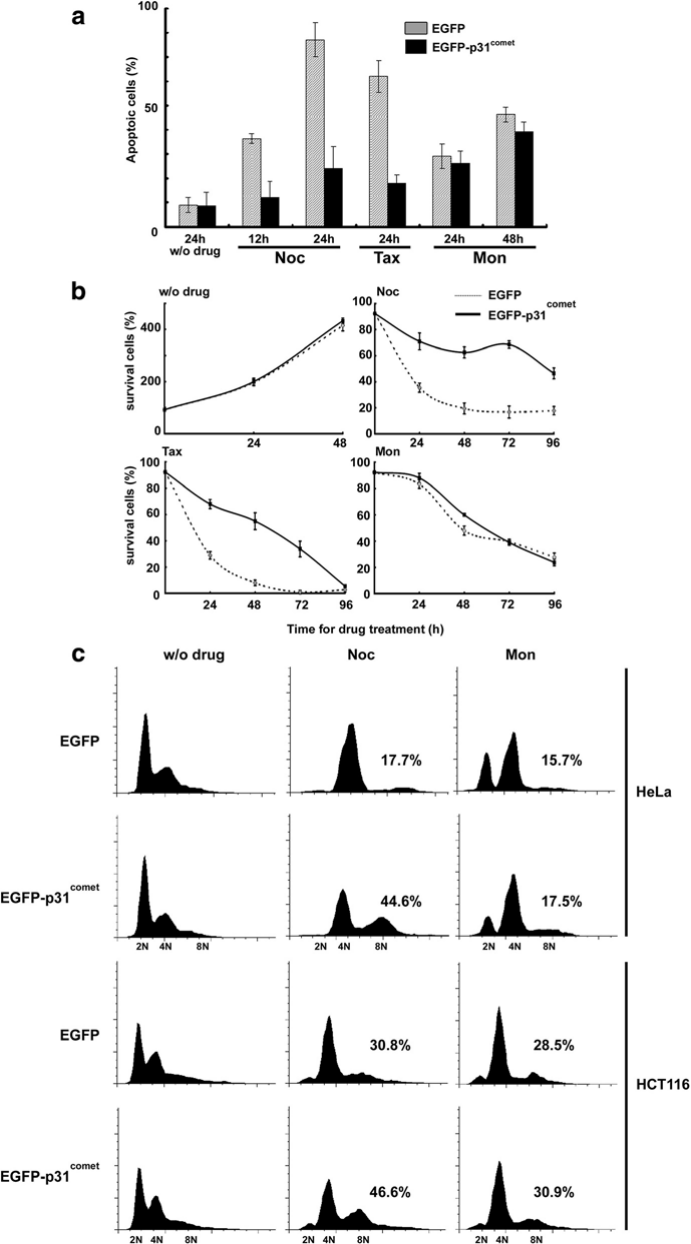
**The effect of p31** ^**comet**^ **overexpression on drug-induced apoptotic cell death. (a)** Apoptosis analysis of p31^comet^-overexpressing cells with continuous treatment by anti-mitotic drugs. HeLa cells overexpressing EGFP or EGFP-p31^comet^ and treated with anti-mitotic drugs were subject to apoptosis analysis with MultiCaspase detection kit. **(b)** Cell survival analysis of p31^comet^ overexpressing cells with continuous treatment by anti-mitotic drugs. HeLa cells overexpressing EGFP or EGFP-p31^comet^ and treated with anti-mitotic drugs were subject to trypan blue exclusion to counte live cells at the indicated time points. **(c)** The FACS analysis of p31^comet^-overexpressing HCT116 cells. HeLa or HCT116 cells were infected with EGFP or EGFP p31^comet^ adenovirus for 24 h, and the viruses were washed out with fresh medium. The infected cells were incubated with or without nocodazole for 24 h and prepared for FACS analysis. A population of >4 N DNA content in the cells was shown.

Taxanes including taxol are used for chemotherapy of several cancers. Taxol can bind to microtubules and suppress the microtubule dynamics; therefore, cells treated with this drug arrest in mitosis, and prolonged arrest with the drug induces apoptosis. In some cases, cells in prolonged arrest eventually escape the mitotic arrest and exit mitosis through adaptation process (Wang et al. 2000). The adapted cells are able to continue growing and become polyploidy cells without apoptosis (Weaver and Cleveland 2005). The adaptation process remains unclear. Because p31^comet^ overexpression accelerated aneuploidy in the presence of anti-mitotic drugs, we examined the effects of p31^comet^ overexpression on resistance to anti-mitotic drugs (Figure 5). HeLa cells that were infected with EGFP or EGFP-p31^comet^ adenoviruses were treated with anti-drugs (100 ng/mL nocodazole, 100 nM taxol, 100 nM monastrol) for 24 or 48 h as indicated in Figure 2, and the apoptotic cells were monitored (Figure 5a). With nocodazole treatment for 48 h, HeLa cells overexpressing p31^comet^ remained viable and exhibited only 24% apoptotic cells compared to 77.1% for apoptotic cells in EGFP overexpressing cells. Similar results were observed with taxol treatment (18% for p31^comet^-overexpression vs. 62% for EGFP-overexpression). Because HeLa cells overexpressing p31^comet^did not override monastrol-induced mitotic arrest (Figures 2 and 4), the EGFP-and p31^comet^-overexpressing cells treated with monastrol underwent apoptosis with the same kinetics (Figure 5a). Next, we examined the effect of the continuous treatment with these drugs. Two hundred cells overexpressing EGFP or EGFP-p31^comet^ were seeded into fresh medium with anti-mitotic drugs, and cell survival was monitored by counting cells that were not stained by trypan blue dye at indicated time points in Figure 5b. In the short treatment experiment, the viability of EGFP-p31^comet^ overexpressing HeLa cells that were treated with nocodazole or taxol was dramatically elevated, but the same observation was not made with monastrol (Figure 4b). These results indicated that p31^comet^-overexpression induced the resistance to microtubule poisons by inactivating the Mad2-dependent SAC directly.

### p53-independent adaptation by p31^comet^ overexpression

Mad2 and p53 double knockout mice and embryonic fibroblast cells were viable and exhibited chromosomal instability (Burds et al. 2005), although Mad2 single knockout mice were lethal (Michel et al. 2001). These studies indicated that p53 protein guarded the chromosomal loss and/or gain with SAC machinery. To address the p53 dependency in aneuploidy from p31^comet^ overexpression, p31^comet^ was overexpressed in HCT116 cells, which are colorectal carcinoma cells, and the p53-dependent checkpoint was functional. Because p53 protein is less or not expressed in HeLa cells compared with normal cells, p31^comet^-overexpressing HeLa cells that were treated with anti-mitotic drugs were able to override SAC like Mad2 and p53 double knockout mice and the cells (Figure 5b and c). Interestingly, p31^comet^-overexpressing HCT116 cells in the presence of nocodazole were also able to lead aneuploidy like HeLa cells with similar kinetics (Figure 5c). H1299 cells, which are non-small cell lung cancer cells do not express p53 protein, were used to overexpress p31^comet^ in the presence of anti-mitotic drugs. Using these cells, the overexpression of p31^comet^ did not override the nocodazole-induced SAC, but it could override taxol-induced SAC (Additional file 1: Figure S5) in a similar manner like HeLa and HCT116 cells. These results suggested that cells overexpressing p31^comet^ in the presence of spindle poisons exit mitosis in a p53-independent adaptation pathway.

### Expression of p31^comet^ in cancer cell lines and resistance against taxol

**Figure 6:**
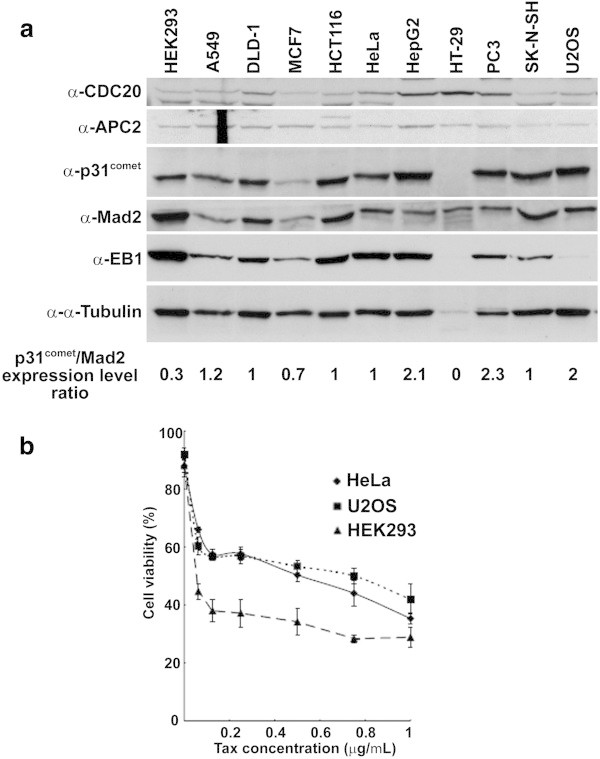
**The effect of p31** ^**comet**^ **expression levels on drug resistance cancer cell lines. (a)** Western blotting analysis of p31^comet^ expression levels in cancer cell lines. Each cell extract was subject to western blotting with anti-p31^comet^ antibody, anti-Mad2 and anti-Cdc20 antibodies to monitor SAC components, anti-EB1 and anti-α-Tubulin antibodies to monitor microtubule components, and anti-APC2 antibody for a loading control. The quantitative p31^comet^/Mad2 expression level ratios were shown. **(b)** Cell viability analysis of cancer cell lines against taxol. Each cancer cell lines with different levels of p31^comet^ was treated with the indicated concentration of taxol and subject to trypan blue exclusion to counte live cells at 2 days. Two hundred cells were scored at each point.

The overexpression of p31^comet^ contributed to aneuploidy and resistance to anti-mitotic drugs. To address p31^comet^ function with respect to drug sensitivity, we observed the expression level of p31^comet^ in several cancer cell lines (Figure 6a). Cycling cells were subjected to western blotting analysis, and monitored the p31^comet^, Mad2, and APC2 protein levels. The protein levels of Mad2 and p31^comet^ in the indicated cell lines showed great variations. Quantitative analysis of the Mad2 and p31^comet^ protein expression was performed using the intensity of the APC2 loading control as a standard. Each protein level was normalized to the expression level in HeLa cells (p31^comet^/Mad2 ratio in HeLa cells = 1). The quantitative p31^comet^/Mad2 expression ratios were shown in Figure 6a. In U2OS, PC3, and HepG2 cells, the p31^comet^/Mad2 expression level ratio was higher than in other cell lines. However, in HEK293 and HT-29 cells, the p31^comet^/Mad2 expression level ratio was lower, although the p31^comet^ signal was not detected in HT-29 cells. In A549, HCT116, DLD-1, MCF7, and SK-N-SH cells, the p31^comet^/Mad2 expression level ratio was equal to in HeLa cells. Using U2OS, HEK293, and HeLa cell lines, we tested the sensitivity to taxol. Consistent with the p31^comet^/Mad2 expression level ratio profile (Figure 6a), HEK 293 cellines were more sensitive to taxol than HeLa and U2OS cells (Figure 6b). U2OS celllines, whicih had a higher p31^comet^ expression level, were more resistant against taxol than HeLa cells at higher concentrations (0.4-1 μg/ml) of taxol (Figure 6b). These results indicated that the p31^comet^/Mad2 expression level ratio may contribute to sensitivity to spindle poisons in cancer cells.

## Discussion

### p31^comet^ can abolish the Mad2-dependent SAC

We previously reported that the p31^comet^-Mad2 complex becomes prominent in mid-mitosis and that the overexpression of p31^comet^ in HeLa cells arrested by nocodazole abrogates the arrest that is maintained by the SAC because of the disappearance of the Cdc20-Mad2 complex (Habu et al. 2002). The position between amino acids 55 and 81 of p31^comet^ might be responsible for binding to Mad2 protein. A fine crystal study (Yang et al. 2007) and Westhorpe et al. (2011) showed that p31^comet^ binds to Mad2 protein through multiple points of interaction and that Q83A and F191A mutations in p31^comet^ can abolish Mad2-p31^comet^ binding. Because p31^comet^ C fragment (81-274aa) contained the Q83 position amino acid and was close to the interaction-surface, which forms a coiled-coil structure, this mutant may disrupt the p31^comet^ structure and, making the mutant unable to override nocodazole-induced SAC. Our preliminary results showed that an L76A/L77A mutant of p31^comet^ could not override the Mad2-dependent SAC and then a fragment of p31^comet^ containing amino acid 1-80, which can bind to Mad2, could override nocodazole-induced SAC (Additional file 1: Figure S1). The 1-81 amino acid fragment of p31^comet^ could bind to Mad2 more strongly than other regions of p31^comet^ by two-hybrid assay. These results indicated that the amino-terminal region of p31^comet^ might maintain the binding to Mad2 and regulate functional structure despite the lack of conservation between other p31^comet^ homologs.

We have shown that the overexpression of p31^comet^ caused premature destruction of Securin and did not accumulate phosphorylated form of Cdc27 despite the presence of anti-mitotic drugs. The abrogation of the arrest maintained by the SAC was observed in cells treated with Hec1 siRNA, which caused Mad2-dependent mitotic arrest, but not AuroraA siRNA (Additional file 1: Figure S6) (Martin-Lluesma et al. 2002; Jiang et al. 2003). An immuno-localization study and ectopic expression of Myc or GFP-tagged p31^comet^ in HeLa and PtK2 cells showed that p31^comet^ was localized on kinetochores during prometaphase to metaphase (Westhorpe et al. 2011). These results indicated that the overexpression of p31^comet^ abolishes the Mad2-dependent SAC in a Mad2-kinetochore localization-dependent manner. Surprisingly, cells overexpressing p31^comet^ cannot exit mitosis in the absence of Eg5 activity through the Mad2-dependent SAC, although the overexpression of p31^comet^ can abolish the Mad2-dependent SAC and monastrol-induced mitotic arrest is abrogated by treatment with Mad2 siRNA. Interestingly, the Mad2 protein regulated the SAC and the normal timing of mitotic progression, but it did not regulate other SAC proteins, including Mad1 and Bub3 (Meraldi et al. 2004). Therefore, we speculated that mitotic progression is monitored by Mad2 protein and that Eg5 function may be required for the coordination of mitosis when p31^comet^ is overexpressed in the cells with functional Mad2 protein and/or normal levels of Mad2. Conversely, mitotic progression is accelerated in the absence of functional Mad2 protein and/or lower levels of Mad2 protein. Moreover, monastrol inhibits Eg5 kinesin function, but not microtubule metabolism, which contrasts with the action of other anti-mitotic drugs. Actually, p31^comet^ overexpression in the absence of anti-mitotic drugs did not show any mitotic errors and aneuploid cells in HeLa cells. siRNA studies showed that in the absence of p31^comet^ in HeLa cells, the metaphase to anaphase transition time was delayed compared to normal mitosis (Westhorpe et al. 2011; Varetti et al. 2011; Jia et al. 2011; Hagan et al. 2011). Under normal microtubule environments, p31^comet^ might be able to override only SAC, but not mitotic spindle organization and progression.

Interestingly, the overexpression of AuroraA kinase overrides SAC, and induces resistance to taxol (Anand et al. 2003), and binds to Cdc20 protein (Farruggio et al. 1999). In *Xenopus*, Eg2 (AuroraA) and Eg5 formed a complex in mitosis (Giet et al. 1999). When AuroraA was depleted by siRNA in HeLa cells, the overexpression of p31^comet^ did not abolish the nocodazole-induced SAC (Additional file 1: Figure S6). My preliminary results showed that p31^comet^ localizes to centrosomes in prophase (data not shown). From these observations, we speculate that p31^comet^ may function with AuroraA kinase and Eg5 kinesin in mitotic events. This indicates that inhibiting Eg5 kinesin function may be useful for cancer therapies of cancer cells that have abbreviations in SAC.

### p31^comet^ overexpression and nocodazole and taxol sensitivity

We showed here that p31^comet^ overexpression caused aneuploidy following the abrogation of a sustained SAC and led to resistance to nocodazole and taxol in HeLa and HCT116 cells. Strikingly, these resistant cells against nocodazole and taxol were also the resistant to apoptotic cell death induced by continuous drug treatment. Interestingly, CDK1 activity is required for promotion of apoptosis after SAC activation with spindle poisons, and the apoptosis occurred after rereplication and abnormal mitosis (Chan et al. 2008). The overexpression of p31^comet^ in HeLa cells arrested by nocodazole abrogates SAC following degradation of cyclinB_1_ and Securin. Collectively, these data indicate that the overexpression of p31^comet^ in human cells shows similar effect with treatment with CDK inhibitors. Chromosomal instability has been thought to be linked to defects in SAC in human cancers and associated with tumorigenesis and/or progression. Increasing evidence has shown that a loss of SAC regulation causes premature exit from mitosis and subsequently leads to chromosomal instability (Weaver and Cleveland 2005). However, alterations in the expression levels in SAC genes were reported in human cancers (Hanks et al. 2004; Cheung et al. 2005; Kienitz et al. 2005; Kim et al. 2005). Furthermore, decreased Mad2 expression level has led to increased chemosensitization to spindle poisons such as vincristine. The p31^comet^ gene locus was mapped to chromosome 6p21.1 and cytogenetic abnormalities of 6p21.1, including amplifications, deletions, and translocations, have been reported in osteosarcoma (Dobles et al. 2000; Hanks et al. 2004; Burds et al. 2005), mature B cell malignancies (Sonoki et al. 2001) and squamous cell carcinoma (Brass et al. 1996; Mazurenko et al. 1999; Wang et al. 2003; Cheung et al. 2005; Jin et al. 2012). Ma et al. reported that the sensitivity to spindle poisons was enhanced and spindle poison-induced cell death was elevated in p31^comet^-depleted HeLa cells. Therefore, we speculate that the expression level of p31^comet^ may contribute to SAC-dependent chromosomal instabilities in human cancers. Furthermore, we showed here that aneuploidy and resistance to spindle poisons caused by the overexpression of p31^comet^ is a p53-independent adaptation pathway in culture cells. Mad2 and p53 double knockout cells can survive, but the same is not true for Mad2 single knockout cells (Dobles et al. 2000; Burds et al. 2005). Using various cancer cell lines, p31^comet^/Mad2 protein expression ratio appears to contribute taxol-resistance (Figure 6). Cdc20 protein expression level was also variable in used cancer cells lines, but Cdc20/Mad2 protein expression ratio seems to be dispensable for taxol resistance compared to p31^comet^/Mad2 ratio. Similar result was indicated that the expression of p31^comet^ and Mad2 correlated with timing of mitotic slippage in various cancer lines (Ma et al. 2012). Therefore, the expression level of p31^comet^ may contribute to chromosomal instabilities in cells with a functional SAC and functional p53 checkpoint machinery at the initial stage of tumorigenesis.

## Methods

### Cell culture, and adenovirus transduction, and siRNA transfection

HeLa, HEK293, 293A, and MCF7 cells were grown under standard conditions in DMEM supplemented with 10% FBS and penicillin and streptomycin. A549, DLD-1, H1299, HCT116, HepG2, HT-29, PC3, SK-N-SH, and U2OS cells were grown under standard conditions in RPMI medium supplemented with 10% FBS and penicillin and streptomycin. N-terminus fused EGFP-Mad2 in the pEGFP-C1 plasmid was introduced into HeLa cells and selected with G418 (Life Technologies, Carlsbad, CA). Cells stably expressing EGFP-Mad2 were confirmed by observation with fluorescence microscopy and western blotting with anti-GFP (Roche Applied Science, Mannheim, Germany) and anti-Mad2 (Covance Inc., Princeton, NJ) antibodies.

Recombinant adenoviruses were produced using the ViraPower adenovirus kit (Life Technologies) according to the manufacturer’s protocol. Amplified recombinant adenovirus was titrated, and the expression of the EGFP-fused gene was monitored. EGFP and EGFP-fused p31^comet^ (full (amino acids 1-274), A (31-274), B (55-274), C (81-274), and D (108-274)) were cloned into the pENTR4 plasmid (Life technologies). These plasmids were recombined with the pAd-CMV/DEST plasmid (Life Technologies) using LR-ClonaseII (Life Technologies). These plasmids were transfected into 293A cells, and recombinant adenovirus was produced. To express EGFP or EGFP-p31^comet^ protein in HeLa cells, 1 × 10^5^ cells were seeded into 6 wells plate before transduction with adenovirus and were incubated 24 h with adenovirus at multiplicity of infection = 5. After the incubation, cells were washed with fresh DMEM containing 10% FBS, and added fresh medium with the indicated concentrations of nocodazole (Merk, Darmstadt, Germany) or taxol (Tocris Bioscience, Bristol, UK) or monastrol (Tocris) was added. After the further incubation for 24 h, cells were collected and analyzed. siRNA duplexes to repress Eg5 (#8100064834, QIAGEN GmbH, Hilden, Germany), control (#1027280, QIAGEN), and Mad2 (#SI02653735, QIAGEN) were transfected using Nucleofector device and transfection reagent (Lonza, Visp, Switzerland) according to the manufacturer’s instructions. In brief, 10^6^ cells were collected and washed with fresh medium. The cells were resuspended in 100 μL transfection reagent, mixed with siRNA duplexes, and transfected with a Nucleofector device. The cells were seeded in wells of a 6-well plate; after 6 h or 12 h, 1.5 × 10^5^ cells were replated in wells of a 6-well plate. Cells were analyzed 24-60 h after transfection.

### Immunoprecipitation and western blotting

Harvested cells were washed once with phosphate buffered saline (PBS, pH 7.2) without calcium and magnesium and lysed in nonidet p-40 (NP-40) lysis buffer (50 mM Hepes, pH 7.4, 250 mM NaCl, 1% NP-40, 5 mM ß-glycerophosphate, 1 mM phenylmethylsulfonyl fluoride, 2 mM Na_3_VO_4_, 0.2 mM ethylenediaminetetraacetic acid (EDTA), 10 mM NaF, 1 mM dithiothreitol and protease inhibitor cocktail (Nacalai, Kyoto, Japan). Cell lysates were incubated at 0°C for 20 min and centrifuged at 8,500 ×g for 15 min. For immunoprecipitation, the supernatants were incubated with anti-GFP antibody conjugated with agarose beads (MBL, Nagoya, Japan) for 4 h at 4°C. The immunoprecipitates were washed once with NP-40 lysis buffer, washed twice with NP-40 lysis buffer without NaCl, and subjected to western blot. Antibodies to p31^comet^ (Habu et al. 2002) or GFP (Roche) were used at a concentration of 0.5 μg/mL. The antibody to Mad2 (Covance) was used at the recommended dilution. Other antibodies, anti-Cdc27 (BD Biosciences, Franklin Lakes, NJ), anti-Cdc20 (Life Technologies), anti-a-Tubulin (Sigma, St. Louis, MO), anti-Eg5 (BD Biosciences), anti-EB1 (BD Biosciences), and anti-Securin and anti-APC2 (Life Technologies), were used at a concentration of 1 μg/mL.

### FACS analysis, apoptosis assay, and cell survival assay

FACS analysis was performed with a standard method, and fluorescence was measured with a Guva PCA instrument (GE Healthcare, Uppsala, Sweden). The apoptosis assay was performed with a Guva MultiCaspase detection kit (GE Healthcare) using a Guva PCA instrument. Dead cells including early to late apoptotic cells and dying cells, were measured to distinguish them from live cells. The survival assay was performed with trypan blue exclusion. EGFP- or EGFP-p31^comet^ overexpressing cells (1 × 10^5^ cells/ sample) were plated in 24 wells dish (2 × 10^2^ cells/well) and treated with each drug for the indicated time. The cells were dislodged and stained with trypan blue dye (Sigma), and the unstained cells were counted for cell survival.

### Cell staining

Cells grown on poly-L-lysine–coated cover slips were washed with PHEM buffer (60 mM Pips, 25 mM Hepes, pH 6.9, 10 mM EDTA, 4 mM MgCl_2_) and permeabilized with 0.2% Triton X-100 in PHEM for 2 min on ice. The cells were fixed with 4% formaldehyde in PHEM buffer for 20 min on ice to visualize EGFP-Mad2. To stain microtubules, the cells were fixed and permeabilized in cold methanol for 2 min. The cells were washed twice with PHEM and blocked with heated goat serum. The cells were incubated with anti-α-tubulin antibody (Sigma) conjugated with Cy3. DNA was visualized with 50 ng/mL of Hoechst 33324 (Nacalai, Kyoto, Japan) for 5 min.

## Electronic supplementary material

Additional file 1: Figure S1: Relationship between the N-terminal region of p31^comet^ and abrogation of the nocodazole induced SAC. **Figure S2.** Monastrol induced Mad2-dependent mitotic arrest. **Figure S3.** Overexpression of p31^comet^ could not override monastrol induced Mad2-dependent mitotic arrest. **Figure S4.** Overexpression of p31^comet^ induced abnormal nuclei and giant cells. **Figure S5.** Overexpression of p31^comet^ could override the taxol-induced SAC, but not Nocodazole-induced SAC in H1299 cells. **Figure S6.** Overexpression of p31^comet^ in Hec1 or AuroraA-knockdown in HeLa cells. (DOCX 410 KB)

## Abbreviations

SAC: Spindle assembly checkpoint
APC: Anaphase promoting complex or cyclosome
O-Mad2: Open Mad2
C-Mad2: Close Mad2
MCC: Mitotic checkpoint complex
RNAi: RNA interference
FACS: Fluorescence-activated cell sorting
PBS: Phosphate buffered saline
NP-40: Nonidet p-40
EDTA: Ethylenediaminetetraacetic acid.
