# Supplementary material for: p31comet inactivates the chemically induced Mad2-dependent spindle assembly checkpoint and leads to resistance to anti-mitotic drugs
Source: Springerplus. 2013 Oct 25;2:562. doi: 10.1186/2193-1801-2-562 (PMC3824705; doi:10.1186/2193-1801-2-562)
Supplement: Supplementary file 1 — Additional file 1: Figure S1: Relationship between the N-terminal region of p31comet and abrogation of the nocodazole induced SAC. Figure S2. Monastrol induced Mad2-dependent mitotic arrest. Figure S3. Overexpression of p31comet could not override monastrol induced Mad2-dependent mitotic arrest. Figure S4. Overexpression of p31comet induced abnormal nuclei and giant cells. Figure S5. Overexpression of p31comet could override the taxol-induced SAC, but not Nocodazole-induced SAC in H1299 cells. Figure S6. Overexpression of p31comet in Hec1 or AuroraA-knockdown in HeLa cells. (DOCX 410 KB) [file 40064_2013_627_MOESM1_ESM.docx]

**Supplementary data**

Title: p31^comet^ inactivates the chemically induced Mad2-dependent spindle assembly checkpoint and leads to resistance to anti-mitotic drugs

Toshiyuki Habu and Tomohiro Matsumoto

Toshiyuki Habu:

Radiation Biology Center, Kyoto University, Yoshida-Konoe cho, Sakyo ku, Kyoto, Japan

E-mail: habu.toshiyuki.6x@kyoto-u.ac.jp

Tomohiro Matsumoto:

Radiation Biology Center, Kyoto University, Yoshida-Konoe cho, Sakyo ku, Kyoto, Japan

Correspondence: Toshiyuki Habu habu.toshiyuki.6x@kyoto-u.ac.jp Phone: +81-75-753-7560, Fax: +81-75-753-7564

**Methods**

**siRNA and antibodies**

siRNA duplexes to repress Hec1 (#SI02653224, QIAGEN), control (#1027280, QIAGEN), and AuroraA (#SI02223305, QIAGEN) were transfected using a Nucleofector device and transfection reagent according to the manufacturer’s instructions. To monitor the knockdown efficiency, western blotting was performed with anti-Hec1 (Abnova, Taipei City, Taiwan) or anti-AuroraA (BD bioscience) antibody.

**Supplemental Fig. 1 Relationship between the N-terminal region of p31^comet^ and abrogation of the nocodazole induced SAC.**

**(a)** An amino-terminal region mutant of p31^comet^ could not bind to Mad2 protein. L76A/L77A (L76/77A) mutations were introduced into full-length EGFP-p31^comet^ cDNA. HeLa cells were infected with adenovirus (EGFP, EGFP-p31^comet^ wt, and EGFP-p31^comet^ L76/77A) and treated with nocodazole for 24 h. Total protein lysates were immunoprecipitated using anti-GFP antibody and subjected to western blotting analysis with anti-Mad2 and anti-GFP antibodies. **(b)** The FACS analysis of p31^comet^ L76/77A mutant or amino-terminal region fragment (1–80 amino acids) of p31^comet^-overexpressing cells treated with nocodazole. The cells were prepared for FACS analysis.

**Supplemental Fig. 2 Monastrol induced Mad2-dependent mitotic arrest.**

The FACS analysis of monastrol-treated cells in which Mad2 was depleted with siRNA. Cells were treated with control or Mad2 siRNA for 24 h and exposed to monastrol at the indicated concentrations for 24 h. The cells were prepared for FACS analysis. A population of >4N DNA content in the cells was shown.

**Supplemental Fig. 3 Overexpression of p31^comet^ could not override monastrol induced Mad2-dependent mitotic arrest.**

The FACS analysis of p31^comet^ overexpressing cells treated with both nocodazole and monastrol. Cells were infected with EGFP or EGFP-p31^comet^ adenovirus for 24 h and the cells were washed with fresh medium twice. The cells were treated with nocodazole at the indicated concentration for 6 h and, treated with monastrol for18 h. These treated cells were prepared for FACS analysis. A population of >4N DNA content in the cells was shown.

**Supplemental Fig. 4 Overexpression of p31^comet^ induced abnormal nuclei and giant cells.**

**(a)** Chromosome analysis of p31^comet^ overexpressing cells. Cells were infected with EGFP or EGFP-p31^comet^ adenovirus for 24 h, and the cells were washed with fresh medium twice. The cells were treated with nocodazole or taxol for 24 h. The treated cells were fixed in methanol/acetone solution and spread onto glass slides. The chromosome spreads were stained with Giemsa dye and observed by microscopy. **(b)** Light-microscopy analysis of p31^comet^-overexpressing cells. Cells were infected with EGFP or EGFP-p31^comet^ adenovirus for 24 h, and the cells were washed with fresh medium twice. The cells were treated with nocodazole or taxol for 24 h and directly observed by microscopy.

**Supplemental Fig. 5 Overexpression of p31^comet^ could override the taxol-induced SAC, but not Nocodazole-induced SAC in H1299 cells.**

The FACS analysis of p31^comet^ overexpressing H1299 cells treated with both nocodazole and taxol. Cells were infected with EGFP or EGFP-p31^comet^ adenovirus for 24 h, and the cells were washed with fresh medium twice. The cells were treated with nocodazole or taxol for 24 h. These treated cells were prepared for FACS analysis. A population of >4N DNA content in the cells was shown.

**Supplemental Fig. 6 Overexpression of p31^comet^ in Hec1 or AuroraA-knockdown in HeLa cells.**

**(a)** Western blotting analysis of p31^comet^-overexpressing cells with Hec1-depletion. The siRNA-treated cell extracts were subject to western blotting with anti-Hec1 antibody to monitor Hec1 depletion, anti-Securin antibody to monitor cell cycle status, anti-GFP antibody for EGFP and EGFP-p31^comet^, and anti-Mad2 antibody for a loading control.

**(b)** FACS analysis of p31^comet^overexpressing cells with Hec1-depletion. Cells treated with Hec1 siRNA or control siRNA were infected with EGFP or EGFP-p31^comet^ adenovirus and prepared for FACS analysis.

**(c)** FACS analysis of p31^comet^ overexpressing cells with AuroraA-depletion. Cells treated with AuroraA siRNA or control siRNA were infected with EGFP or EGFP-p31^comet^ adenovirus and prepared for FACS analysis.
